# A Case of Malignant Lymphoma With Cerebral Intravascular Component Diagnosed at Autopsy With Repeated Stenosis of the Bilateral Anterior Cerebral Artery

**DOI:** 10.7759/cureus.83660

**Published:** 2025-05-07

**Authors:** Genki Ikuta, Naohiro Taura, Daisuke Muta

**Affiliations:** 1 Neurosurgery, Hitoyoshi Medical Center, Kumamoto, JPN; 2 General Medicine, Hitoyoshi Medical Center, Kumamoto, JPN

**Keywords:** anterior cerebral artery, hemorrhagic stroke, intravascular large b-cell lymphoma, ischemic stroke, magnetic reasoning imaging, malignant lymphoma

## Abstract

Intravascular large B-cell lymphoma (IVLBCL) is a rare disease characterized by selective proliferation of lymphoma cells within the lumen of blood vessels, especially capillaries. When IVLBCL involves the central nervous system (CNS), it could cause a stroke. IVLBCL is often difficult to diagnose because the disease lacks specific imaging findings and symptoms and can sometimes progress rapidly. We report a rare case of IVLBCL with repeated stenosis and improvement of the bilateral anterior cerebral artery (ACA) that required a long time to diagnose. The patient was admitted to our hospital three times (days 1-22, days 126-171, and days 212-221) after ischemic stroke. After acute-phase treatment, the patient was transferred to a rehabilitation hospital but developed systemic symptoms such as diarrhea, dizziness, and vomiting with ischemic stroke and was transferred back to our hospital. Various examinations were performed, but the causes remained unknown. Malignant lymphoma was strongly suspected at the third hospitalization; however, before treatment was started, the patient died of a hemorrhagic stroke. An autopsy revealed a relatively large number of lymphoma cells in the small vessels of the subarachnoid space of the brain, consistent with IVLBCL. Because of repeated systemic symptoms and dynamic evolution of ischemic stroke with repeated stenosis of the bilateral ACA, some diseases were suspected to be the etiology of stroke; however, diagnosis was not made until the patient died, and follow-up was delayed because IVLBCL progressed aggressively. In addition to the aggressive and atypical clinical course, imaging findings of repeated stenosis of the bilateral ACA may help suspect IVLBCL.

## Introduction

Intravascular large B-cell lymphoma (IVLBCL) is a rare disease characterized by selective proliferation of lymphoma cells within the lumen of blood vessels, especially capillaries [1]. Clinical signs and symptoms vary depending on the target organ. When IVLBCL involves the central nervous system (CNS), it could cause a stroke. CNS symptoms of IVLBCL are nonspecific, including cognitive impairment, dementia (60.9%), paralysis (22.2%), and seizures (13.4%) [2]. IVLBCL sometimes progresses rapidly; if not diagnosed and treated early, patients with IVLBCL cannot survive for >1 year. In >50% of the cases, the diagnosis is made incidentally during autopsy [2]. Herein, we present the case of a 59-year-old woman with an unusual course of repeated stenosis of the bilateral anterior cerebral artery (ACA), who died of cerebral hemorrhage. Autopsy findings revealed lymphoma cells in the small vessels of the subarachnoid space of the brain, which is consistent with IVLBCL.

## Case presentation

Days 1-22: Initial presentation and management

A 59-year-old woman with right lower limb paralysis of three days duration presented to the emergency department. The patient had no relevant family or medical history, allergies, or smoking history. Neurological examination revealed a Glasgow Coma Scale score of 15, mild dysarthria, and paralysis of the right upper limbs (manual muscle testing [MMT] grade 4) and lower limbs (MMT 1).

The results of the blood test were as follows: white blood cell count (WBC), 5,600/µL; hemoglobin (Hb), 13.8 g/dL; platelet count (Plt), 214,000/µL; albumin (Alb), 4.4 g/dL; lactate dehydrogenase (LDH), 286 U/L; estimated glomerular filtration rate (eGFR), 75.5 mL/min/1.73 m^2^; low-density lipoprotein (LDL), 168 mg/dL; hemoglobin A1C, 6.0% (Table 1).

**Table 1:** Laboratory parameters of the patient during the different times of the follow-up H: higher than the reference range; L: lower than the reference range.

Lab parameter	During the first hospitalization	During the second hospitalization	During the third hospitalization	Reference values
White blood cell count (/µL)	5,600	4,100	4,900	3,300-8,600
Hemoglobin (g/dL)	13.8	12.8	12.9	11.6-14.8
Platelet count (×10^3^/µL)	214	229	283	158-348
Albumin (g/dL)	4.4	2.7 (L)	1.6 (L)	4.1-5.1
Lactate dehydrogenase (U/L)	286 (H)	240 (H)	385 (H)	124-222
Estimated glomerular filtration rate (mL/min/1.73 m^2^)	75.5 (L)	83.9 (L)	46.2 (L)	>90
Low-density lipoprotein (mg/dL)	168 (H)			65-163
Hemoglobin A1C (%)	6.0			4.9-6.0
D-dimer (µg/mL)	0.0	3.0 (H)	1.1 (H)	<1.0
Ferritin (ng/mL)		298 (H)		3.6-114
Soluble interleukin-2 receptor (U/mL)		1340 (H)	3109 (H)	157-474
Antinuclear antibodies		Negative		
Anti-Sm antibodies		Negative		
Anti-double-stranded DNA antibodies		Negative		
Anti-RNA polymerase III antibodies		Negative		
Anti-ribonucleoprotein antibodies		Negative		
Anti-Scl-70 antibodies		Negative		
Anti-centromere antibodies		Negative		
Lupus anticoagulant		Negative		
Cytoplasmic antineutrophil cytoplasmic antibodies		Negative		
Perinuclear antineutrophil cytoplasmic antibodies		Negative		

**Figure 1:**
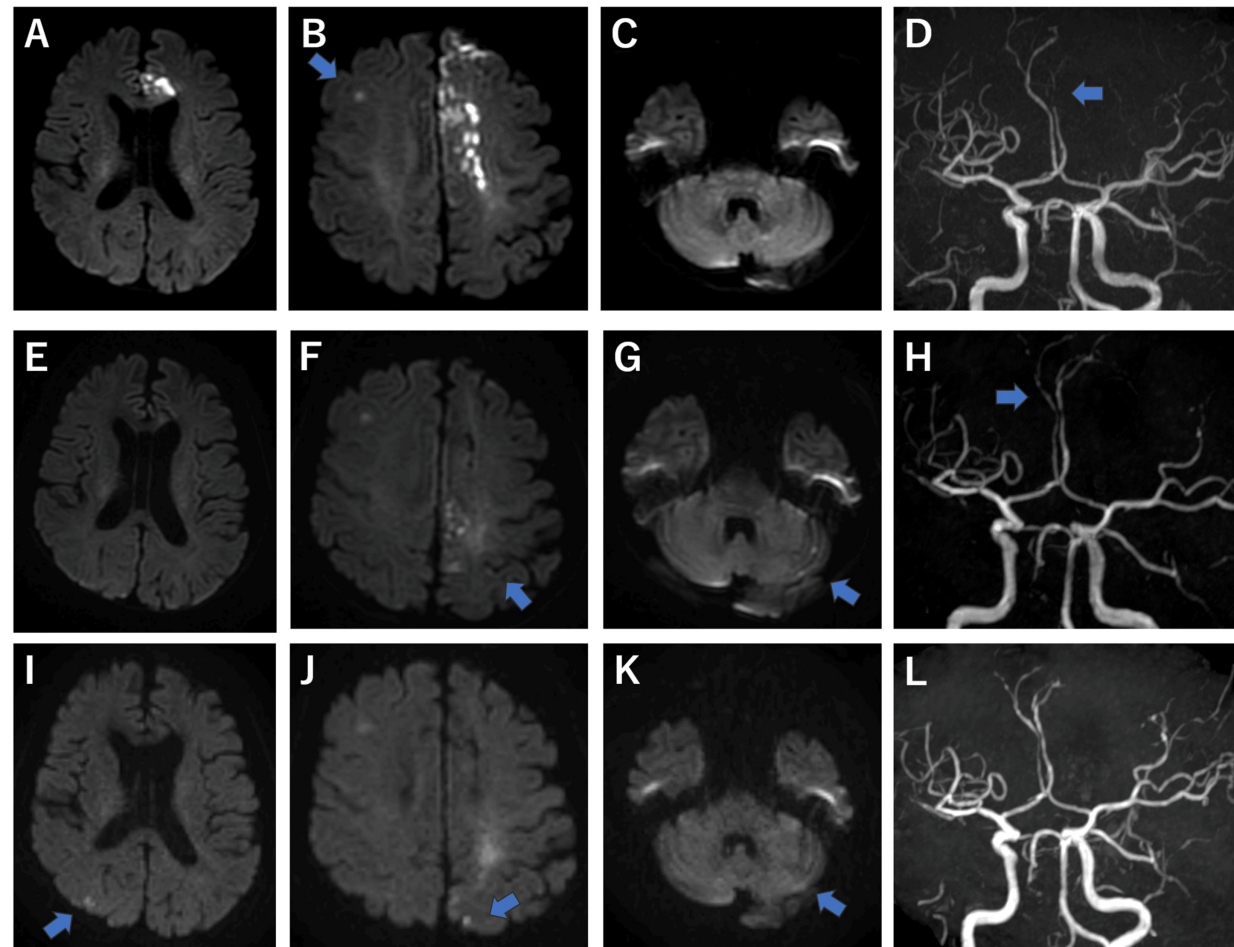
Time course of brain magnetic resonance imaging (MRI) Time course of brain magnetic resonance imaging: (A-D) During the first hospitalization (day 1). (E-H) During the second hospitalization (day 126). (I-L) During follow-up at the second hospitalization (day 139).

Brain magnetic resonance imaging (MRI) revealed stenosis of the left ACA and acute ischemic stroke in the left ACA, partly outside the area with hyperintensity on diffusion-weighted and T2-weighted images (Figure 1). Contrast-enhanced computed tomography (CT) angiography revealed severe stenosis of the left ACA and no dissection of the left ACA or irregular plaques of the bilateral carotid arteries and aortic arch. Holter electrocardiography showed five consecutive premature atrial contractions, and transesophageal echocardiography showed no patent foramen ovale. In addition to ACA stenosis, sparse cerebral infarctions were observed in multiple cerebral vascular territories. Therefore, cardioembolic stroke or atherothrombotic cerebral infarction was suspected, and rivaroxaban and statins were initiated. The patient was transferred to a rehabilitation hospital on day 22 of hospitalization. The patient developed diarrhea, paroxysmal dizziness, and vomiting on day 110 and was transferred to our department for further examination on the 126th day.

Days 126-177: Presentation of systemic symptoms and recurrence of stroke 

**Figure 2:**
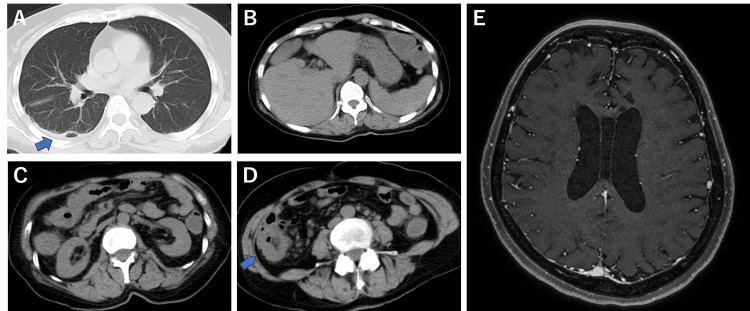
Imaging tests during the second hospitalization (days 126-171) (A) Pulmonary computed tomography (CT). (B-D) Abdominal CT. (E) Contrast-enhanced magnetic resonance imaging (MRI).

During the second hospitalization, the vital signs were temperature, 37.2°C; blood pressure, 120/76 mmHg; heart rate, 80 bpm; and oxygen saturation, 98% room air. Neurological examination revealed no anisocoria, mild nystagmus on the right side, slow, but not slurred speech, or hypoesthesia in the right upper and lower limbs. Barre’s test showed mild pronation of the right upper limb, whereas Mingazzini’s test showed mild agitation of the right lower extremity. A systemic examination was conducted, considering the possible diagnosis of Trousseau syndrome or complications of vasculitis, brain tumor, autoimmune disease, and malignant lymphoma. Brain MRI revealed subacute infarctions in the left parietal lobe and the left cerebellar hemisphere, with partial improvement in the stenosis of the left ACA and the appearance of a new stenosis of the right ACA (Figure 1). Gadolinium-enhanced T1 showed no noticeable enhancement in the parenchyma, pia mater, or dura mater (Figure 2). Pulmonary CT showed cord-like and linear shadows in the lower lobe of the right lung, which were suspected to be due to residual inflammatory changes. Abdominal CT revealed mild splenomegaly and colonic wall thickening, without lymphadenopathy or increased mesenteric fat attenuation (Figure 2). Blood test results were WBC, 4,100 /µL; Hb, 12.8 g/dL; Plt, 229,000/µL; Alb, 2.7 g/dL; LDH, 240 U/L; eGFR, 83.9 mL/min/1.73 m^2^; D-dimer, 3.0 µg/mL; ferritin, 298 ng/mL; and soluble interleukin-2 (IL-2) receptor, 1,340 U/mL. LDH decreased compared to the initial presentation, and soluble IL-2 receptor was mildly high; it was too weak to suspect malignant lymphoma actively. Normal protein S, protein C, and lupus anticoagulants ruled out thrombophilia. Antibodies (antinuclear, cytoplasmic antineutrophil cytoplasmic, perinuclear antineutrophil cytoplasmic, anti-Sm, anti-double-stranded DNA, anti-RNA polymerase III, anti-ribonucleoprotein, anti-Scl-70, and anti-centromere) were negative (Table 1). Tumor marker levels were within normal limits. On day 130, she developed enteritis with sepsis and was started on meropenem, which was later de-escalated to ceftriaxone after blood and stool cultures tested positive for *Klebsiella oxytoca*. Paralysis progressed to MMT3 in the left upper limb and MMT1 in the left lower extremity, and clopidogrel was added. Altered consciousness with occasional rightward conjugate eye deviation and right upper limb spasms were occasionally observed on day 134; thus, an antiepileptic drug was started. MRI on day 139 showed improvement in the right ACA; however, new acute ischemic strokes in both cerebral hemispheres and the left cerebellar hemisphere were observed (Figure 1). Abdominal CT on day 140 showed that the edema in the colon had disappeared. Bone marrow aspiration and gastrointestinal endoscopy were performed due to mild abnormalities in the spleen and colon; however, no abnormalities were detected. The vomiting and diarrhea improved; hence, the patient was transferred to a rehabilitation hospital on day 171. Vomiting recurred on day 212, and the patient was transferred to our hospital's Department of General Medicine for further examination.

Days 212-221: Progression of systemic symptoms and revelation of malignant lymphoma

**Figure 3:**
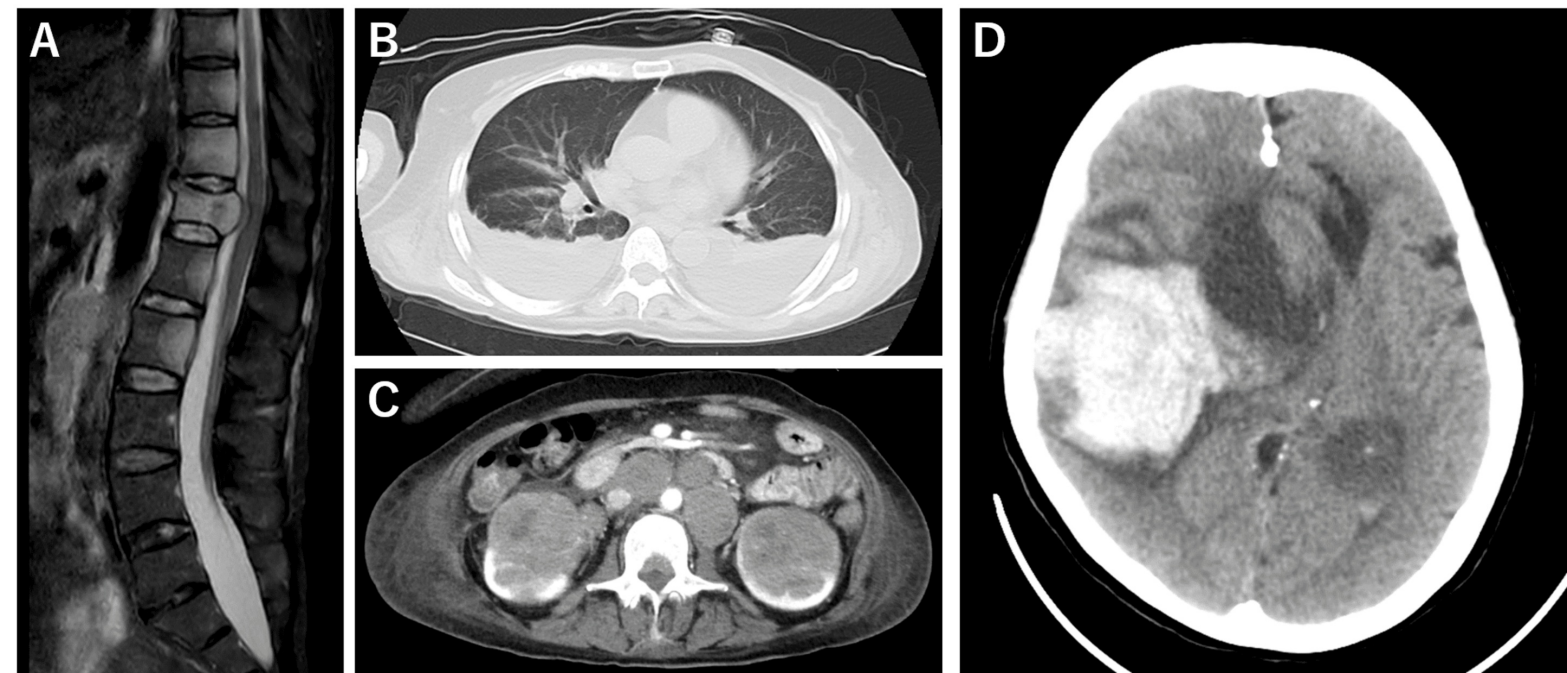
Imaging tests during the third hospitalization (days 212-221) (A) Spine magnetic resonance imaging (MRI). (B) Pulmonary computed tomography (CT). (C) Contrast-enhanced abdominal CT. (D) Brain CT performed after sudden loss of consciousness.

During the third hospitalization, blood test showed the following results: WBC, 4,900/µL; Hb, 12.9 g/dL; Plt, 283,000/µL; Alb, 1.6 g/dL; LDH, 385 U/L; eGFR, 46.2 mL/min/1.73 m^2^; and soluble IL-2 receptor, 3,109.0 U/mL (Table 1). The patient was able to eat slightly better; she was diagnosed with moderate depression and apathy and developed lower back pain. Spinal MRI was performed on day 219, which showed multiple masses in the thoracolumbar spine and bilateral kidneys and multiple enlarged lymph nodes in the para-aortic area. Contrast-enhanced CT revealed a hypovascular renal tumor, and a malignant lymphoma was suspected (Figure 3). On the evening of the same day, she experienced sudden vomiting followed by impaired consciousness with anisocoria. Brain CT revealed an extensive cerebral hemorrhage with a midline shift in the right cerebral hemisphere. The patient died on day 221 (Figure 3).

Results of the autopsy 

Tumor cells were identified at multiple anatomical sites, including the para-aortic and left renal hilum lymph nodes, bilateral kidneys, thoracic and lumbar vertebrae, uterine body, right ovarian hilum, spinal dura mater, and small vessels in the subarachnoid space of the brain, cerebral parenchyma, and lungs. 

**Figure 4:**
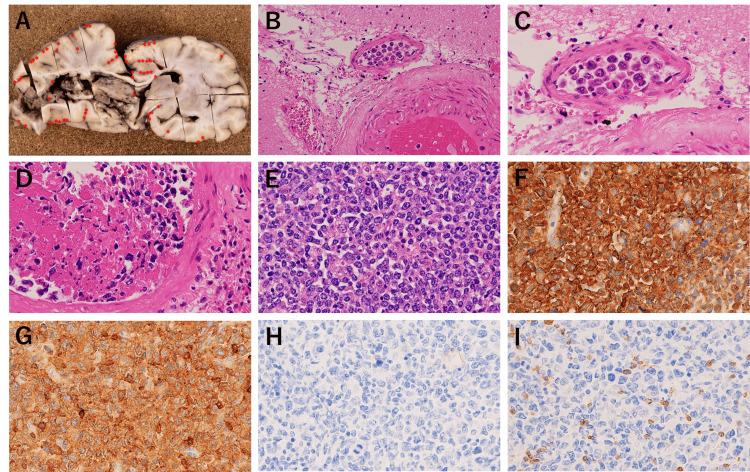
(A) Macroscopic findings of brain at autopsy, (B-D) hematoxylin and eosin staining, and (E-I) immunohistochemical staining of the paraaortic lymph node (×400) (A) Lymphoma cells (*) distributed in the subarachnoid space of the brain and hemorrhage found in the right hemisphere. (B) Diffuse lymphoid infiltration was observed in the non-hemorrhagic lesion in the left cerebral hemisphere; ×100. (C) ×400. (D) Tumor cells were not confirmed in the blood vessels of the hemorrhagic lesion in the right cerebral hemisphere; ×400. (E) Effacement of normal architecture by neoplastic lymphoid cells was observed in paraaortic lymph nodes. (F) CD20: diffuse positive. (G) CD5: aberrant co-expression in tumor cells. (H) CD10: negative. (I) CD3: negative.

Immunohistochemical analysis demonstrated that the tumor cells were positive for CD5, CD20, and Bcl-2 but negative for CD3, CD10, CD30, and cyclin D1 (Figure 4). Tumor cells were not confirmed in the blood vessels of the cerebral hemorrhage lesion or around the bilateral ACA. Arteriosclerotic changes were observed in some parts of the ACA. IVLBCL was possible because no lymphoma was detected on previous imaging, bone marrow aspiration, or gastrointestinal biopsy. Additionally, a relatively large number of lymphoma cells were present in the small vessels of the subarachnoid space of the brain, with a small number of lymphoma cells in the small vessels in the cerebral parenchyma, different from the typical IVLBCL because of the enlargement of the para-aortic and left renal hilum lymph nodes. The patient was diagnosed with either IVLBCL or diffuse large B-cell lymphoma (DLBCL) with intravascular components.

## Discussion

IVLBCL was first described in the third edition of the World Health Organization (WHO) classification system. According to the fourth edition, lymphoma cells in IVLBCL are characterized by selective proliferation in the lumen of blood vessels, especially in capillaries, and minimal extravascular proliferation may be observed. IVLBCL is generally considered to lack lymphadenopathy but may be observed in some patients. The definition of IVLBCL was not changed in the fifth edition of the WHO classification [1].

IVLBCL appears to constitute a distinct group within DLBCL, with features that extend beyond its broad clinical manifestations and phenotypic heterogeneity of CD5 and CD10. The frequency of CD5 expression in IVLBCL varies from 22% to 75%. No significant difference was noted in prognosis between patients with CD5+ and CD5- IVLBCL. However, CD5+CD10- tumors are significantly associated with higher frequencies of thrombocytopenia, bone marrow, and peripheral blood involvement, and lower frequencies of neurologic abnormalities than the CD5-CD10- type. CD5+CD10- IVLBCL and de novo CD5+CD10- DLBCL have much in common, but CD5+CD10- IVLBCL is more strongly associated than CD5+CD10- DLBCL with the following features and parameters: B symptoms, bone marrow involvement, splenomegaly, hepatomegaly, and absence of nodal presentation [3].

IVLBCL obstructs the arterial blood supply to distal locations, causing ischemia in various organs and resulting in systemic symptoms. Clinical signs and symptoms can vary depending on the target organ [2]. CNS complications are common among IVLBCL patients. CNS symptoms were observed in 42% of IVLBCL cases, including cognitive impairment, dementia (60.9%), paralysis (22.2%), and seizures (13.4%) [2].

The usefulness of random skin and liquid biopsies for diagnosing IVLBCL has been reported recently. The former involves skin biopsies from three separate areas, such as the abdomen without a rash, using a scalpel to the depth of the fat-containing areas. False-negative findings are common because of small tumor cell counts. The latter involves checking for cell-free DNA in the peripheral blood, which helps identify tumor-specific mutations, such as MYD88 and CD79B [4,5]. Elevated LDH and soluble IL-2 receptor are significant findings suggestive of IVLBCL; however, the elevated levels of LDH imply rapid cell turnover, and the level of soluble IL-2 receptor, which is found on the lymphocyte membranes, is often elevated in nonhematological diseases, including collagen diseases, solid tumors, and infectious diseases; therefore, their sensitivity and specificity are not high [6].

Differential diagnosis of repeated stenosis and improvement of arteries includes various types of vasculitis and non-inflammatory vasculopathies, including atherosclerosis, vasospasm, radiation vasculopathy, infections, neoplasia, atrial myxomas, neurofibromatosis, and fibromuscular dysplasia. In particular, patients with symptoms or serological markers of systemic inflammation should be considered for infection, malignant neoplasms, or secondary involvement of the CNS as part of systemic vasculitis [7]. According to a report from the National Cerebral and Cardiovascular Center in Japan, the percentage of ischemic stroke with an isolated ACA territory infarction is low (1.3%), whereas cerebral artery dissection accounts for a high proportion (43%) [8]. IVLBCL is usually associated with multiple cerebral infarctions, mostly in subcortical regions, and can show dynamic evolution and resolution of lesions [6,9]. In recent years, the most common lesions in IVLBCL have been reported to be hyperintense lesions in the pons on T2-weighted images (57.6%), caused by local venous congestion and demyelination [10]. Other findings of IVLBCL include nonspecific periventricular white matter lesions due to sluggish flow within the lumens of capillaries or microinfarcts, meningeal enhancement due to an inflammatory reaction with lymphoma cells, mass-like lesions due to the extravasation of lymphoma cells and thickening of the vessel wall, and infarct-like lesions due to vascular occlusion by lymphoma cells [11].

The first case of cerebral hemorrhage complicated by IVLBCL with no involvement of disseminated intravascular coagulation was reported in 2010. The proposed mechanism of hemorrhage includes chronic degenerative or inflammatory changes in the vessel wall, hyalinization, fibrosis, and fibrinoid necrosis due to the direct interaction between atypical lymphoid and endothelial cells of the cerebral capillaries, small arteries, arterioles, and venules [12].

## Conclusions

IVLBCL is an essential etiology of stroke and presents with various imaging findings and symptoms. It can progress rapidly and be fatal if not diagnosed and treated early. IVLBCL should be considered as a differential diagnosis when patients show rapid progression of CNS symptoms and systemic symptoms of unknown cause, and when imaging findings of the CNS show dynamic evolution and resolution of lesions with atypical imaging, including vasculopathy-like findings such as repeated stenosis of the bilateral ACA. The sensitivity and specificity of LDH and soluble IL-2 receptor to IVLBCL are not high; therefore, careful follow-up is required. If IVLBCL is suspected, an intense investigation, including random skin and liquid biopsies, is needed.
